# A Case of Overlooked Popliteal Artery Entrapment Syndrome

**DOI:** 10.7759/cureus.4252

**Published:** 2019-03-14

**Authors:** Lisa Saa, Peter K Firouzbakht, Mohammad Otahbachi

**Affiliations:** 1 Internal Medicine, Texas Tech University Health Sciences Center, Lubbock, USA; 2 Surgery, Texas Tech University Health Sciences Center, Lubbock, USA; 3 Cardiology, Covenant Medical Center, Lubbock, USA

**Keywords:** popliteal artery entrapment syndrome, lower extremity pain, claudication, angiography, paes

## Abstract

Popliteal artery entrapment syndrome (PAES) is an uncommon cause of lower extremity claudication that is often overlooked. It most commonly occurs in young athletes without risk factors for peripheral vascular disease. We present a case of a 47-year-old man who went undiagnosed for over 10 years despite multiple orthopedic, chiropractic, and neurosurgery consults. A definitive diagnosis of PAES was confirmed in the catheterization lab by angiography. The patient underwent popliteal artery bypass surgery and his symptoms completely resolved. PAES must be considered in the differential diagnosis of lower extremity pain, especially in younger patients.

## Introduction

The popliteal fossa is the region of the lower extremity posterior to the knee. It is surrounded by the medial head of the gastrocnemius (inferomedial), the lateral head of the gastrocnemius and plantaris (inferolateral), semitendinosus and semimembranosus (superomedial), and biceps femoris (superolateral) muscles. This fossa contains the popliteal artery, popliteal vein, and tibial nerve. Of the three, the artery is most medial while the tibial nerve is most lateral. The popliteal artery is an extension of the femoral artery. While in the fossa, the popliteal artery is most medial; prior to entering the fossa, the femoral artery is lateral to the femoral vein [1].

Anatomic popliteal artery entrapment syndrome (PAES) is a rare congenital abnormality. Atypical embryological development of the popliteal fossa can result in compression of the popliteal artery in any one of four ways: the artery courses medially around the gastrocnemius (type I), lateral attachment of the medial head of the gastrocnemius (type II), an accessory slip of gastrocnemius muscle (type III), or the artery running below the popliteus muscle or a fibrous band of the popliteus muscle (type IV). Functional PAES (type VI) similarly leads to the occlusion of the artery due to a suspected etiology of hypertrophy of the gastrocnemius muscle, without an obvious anatomic abnormality in the popliteal fossa [2-4].

Most studies report a higher occurrence in male athletes with a mean age between 30 and 35 and find that it is often bilateral [5-10]. PAES may be asymptomatic or it may cause lower extremity claudication, coldness, and numbness during activity. In contrast to peripheral vascular disease where the pain is classically alleviated with rest, the pain in PAES can linger despite rest [5-6].

## Case presentation

A 47-year-old Caucasian male with a past medical history of hypertension, hyperlipidemia, and everyday tobacco use presented with left lower extremity pain for over 10 years. The pain, described as a throbbing and cramping sensation without immediate relief after rest, had worsened significantly over the past two years, leading him to seek further medical attention. Prior to the onset of lower extremity pain, the patient was healthy and led an active lifestyle. On exam, his feet were warm and pink with normal sensation and 2+ distal pulses.

**Figure 1:**
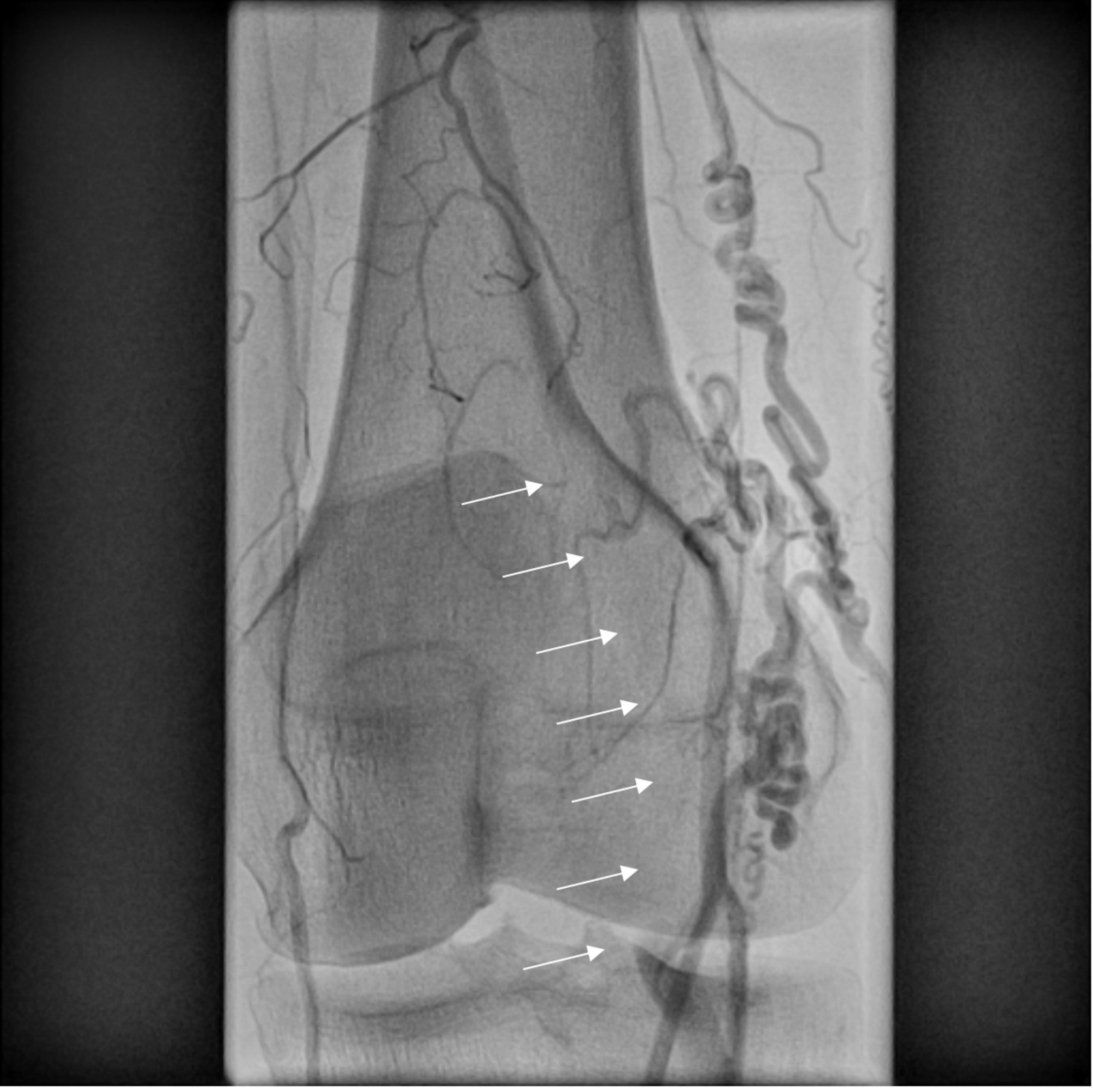
Left lower extremity popliteal artery exhibiting significant collateral arteries. Arrows depict popliteal artery occlusion without vascular flow.

Arterial Doppler of the left lower extremity noted severe stenosis of the left superficial femoral artery and popliteal artery. He was started on cilostazol 100 milligrams two times per day, but his symptoms did not improve. Arteriography revealed normal iliac, common femoral, and superficial femoral arteries without atherosclerosis. There was occlusion of the left popliteal artery and extensive collateral flow to the leg. This raised suspicion for PAES and provocative maneuvers were performed during the study. Dorsiflexion and plantar extension of the ankle caused the cessation of the collateral and distal popliteal artery flow. When the ankle was returned to neutral position, the occlusion ceased and flow was restored. These findings confirmed the diagnosis of PAES with resulting popliteal artery occlusion (Figure 1, Video 1). A popliteal artery bypass with a reverse great saphenous vein was performed, and the patient’s symptoms resolved.

**Video 1:** Angiography illustrating the patient performing a provocative maneuver with cessation of the collateral and distal popliteal artery flow. This is followed by the neutral position, at which time restoration of flow is seen.

## Discussion

Popliteal artery entrapment syndrome (PAES) is a potential cause of overlooked intermittent lower extremity pain in patients without atherosclerotic risk factors, especially in young, active males. Despite multiple consultations, the patient was not diagnosed with this syndrome for over 10 years, leading to occlusion of his left popliteal artery. Notably, the patient was an athlete and began to experience symptoms at age 32, consistent with the typical demographic of a 30 to 35-year-old athletic male. We believe the delay in diagnosis to be due to the patient's history of hypertension, hyperlipidemia, and tobacco use, which suggested peripheral vascular disease as a more likely diagnosis.

PAES typically presents in younger men as bilateral lower extremity pain that is not alleviated immediately with rest [5-6]. The physical exam is typically within normal limits [5,11]. However, if it is suspected, the patient should be assessed by the positional stress test, which consists of performing the provocative maneuvers of dorsiflexion or plantar extension of the ankle joint. Typically, the pedal pulse will disappear during these maneuvers if PAES is present. Angiography, computed tomography (CT) angiography, Doppler ultrasound, or magnetic resonance imaging (MRI) may then be used to confirm the diagnosis of PAES. MRI is the preferred method to visualize the popliteal fossa; however, uncertainty still remains regarding the best method for diagnosis [5,8,11]. In addition to PAES, other non-atherosclerotic causes of lower extremity ischemic pain include cystic adventitial disease of the popliteal artery, iliac artery endofibrosis, chronic compartment syndrome, and iliac or femoral giant cell arthritis.

Without treatment and quick diagnosis, consistent compression of the popliteal artery can lead to arterial intimal damage and worsening occlusion [5,11]. The sequelae of long-term PAES include worsening peripheral vascular disease, intramural hematoma or thrombus, stenosis, occlusion, post-stenotic dilation or aneurysm formation, or limb ischemia requiring amputation [2,5-13]. Treatment requires decompression of the entrapping muscle and release of the popliteal artery in order to allow for revascularization, which is typically performed through surgery [2,5-9,11]. For most patients, the graft remains patent after surgery, with five-year patency exceeding 90% [7].

## Conclusions

Popliteal artery entrapment syndrome (PAES) should be considered primarily in patients without atherosclerotic risk factors, especially young athletes who present with lower extremity exertional pain that lingers after rest. However, a medical history that places a patient at risk for peripheral vascular disease should not solely exclude PAES from the differential diagnosis. The diagnosis can be definitively confirmed with arteriography while the patient is performing provocative maneuvers. Surgical decompression of the artery is the preferred treatment of choice for this syndrome.

## References

[1] Netter F (2014). Atlas of Human Anatomy. http://captionpdf.info/atlas-of-human-anatomy-free-books-great-authors-frank-h-netter.pdf.

[2] Gokkus K, Sagtas E, Bakalim T, Taskaya E, Aydin AT (2014). Popliteal entrapment syndrome. A systematic review of the literature and case presentation. Muscles Ligaments Tendons J.

[3] Delaney TA, Gonzalez LL (1971). Occlusion of popliteal artery due to muscular entrapment. Surgery.

[4] Pillai J (2008). A current interpretation of popliteal vascular entrapment. J Vasc Surg.

[5] Liu Y, Sun Y, He X, Kong Q, Zhang Y, Wu J, Jin X (2014). Imaging diagnosis and surgical treatment of popliteal artery entrapment syndrome: a single-center experience. Ann Vasc Surg.

[6] Apigian AK, Landry GJ (2015). Basic data underlying decision making in nonatherosclerotic causes of intermittent claudication. Ann Vasc Surg.

[7] Lejay A, Delay C, Georg Y (2016). Five year outcomes of surgical treatment for popliteal artery entrapment syndrome. Eur J Vasc Endovasc Surg.

[8] Sinha S, Houghton J, Holt PJ, Thompson MM, Loftus IM, Hinchliffe RJ (2012). Popliteal entrapment syndrome. J Vasc Surg.

[9] Corneloup L, Labanère C, Chevalier L (2018). Presentation, diagnosis, and management of popliteal artery entrapment syndrome: 11 years of experience with 61 legs. Scand J Med Sci Sports.

[10] Noorani A, Walsh SR, Cooper DG, Varty K (2009). Entrapment syndromes. Eur J Vasc Endovasc Surg.

[11] Gourgiotis S, Aggelakas J, Salemis N, Elias C, Georgiou C (2008). Diagnosis and surgical approach of popliteal artery entrapment syndrome: a retrospective study. Vasc Health Risk Manag.

[12] Levien LJ, Veller MG (1999). Popliteal artery entrapment syndrome: more common than previously recognized. J Vasc Surg.

[13] Clemens MS, Watson JDB, Scott DJ, Hislop SJ, Heafner TA, Arthurs ZM (2014). Magnetic resonance imaging: occult popliteal artery entrapment syndrome in a young soldier. Ann Vasc Surg.

